# The effect of promoter methylation on *MdMYB1* expression determines the level of anthocyanin accumulation in skins of two non-red apple cultivars

**DOI:** 10.1186/s12870-018-1320-7

**Published:** 2018-06-05

**Authors:** Changqing Ma, Chenjuan Jing, Bo Chang, Jiuying Yan, Bowen Liang, Li Liu, Yazhou Yang, Zhengyang Zhao

**Affiliations:** 1 State Key Laboratory of Crop Stress Biology for Arid Areas, College of Horticulture, Northwest A & F University, Yangling, 712100 Shaanxi China; 2 Shaanxi Research Center of Apple Engineering and Technology, Yangling, 712100 Shaanxi China

**Keywords:** Apple, Pigmentation, Anthocyanin, *MdMYB1* promoter, Methylation

## Abstract

**Background:**

Fruit color in apple (*Malus domestica* Borkh.) is ascribed mainly to the accumulation of anthocyanin pigments, and is an important trait for determining fruit market acceptance. Bagging is a commonly used treatment to enhance the red pigmentation in apple skin. The *MdMYB1* transcription factor gene plays an important role in the biosynthesis of anthocyanin in apple after bag removal, but little is known about how *MdMYB1* transcription is regulated.

**Results:**

In this study, we investigated pigmentation in the non-red skinned cultivars ‘Granny Smith’ and ‘Golden Delicious’ after bag removal. The fruit skins of the two cultivars showed red/pink pigmentation after bag treatment. Transcript levels of *MdMYB1*, the master regulator of anthocyanin biosynthesis in apple, increased, and showed a correlation with anthocyanin content in both cultivars after bag removal. The *MdMYB1* genomic sequences were compared in the two cultivars, which showed that the green-fruited cultivar ‘Granny Smith’ harbors the *MdMYB1–1* and *MdMYB1–2* alleles, while the yellow-fruited cultivar ‘Golden Delicious’ harbors only *MdMYB1–2.* A comparison of methylation levels in the 2 kb region upstream of the *MdMYB1* ATG between the bag-treated fruits after removal from the bags and the unbagged fruits showed a correlation between hypomethylation and the red-skin phenotype in ‘Granny Smith’. Moreover, ‘Granny Smith’ fruits responded to treatment with 5-aza-2′-deoxycytidine, an inducer of DNA demethylation. An investigation of the *MdMYB1* promoter in ‘Granny Smith’ showed reduced methylation in the regions − 2026 to − 1870 bp, − 1898 to − 1633 bp, and − 541 to − 435 bp after bag removal and 5-aza-2′-deoxycytidine treatments.

**Conclusions:**

Differences in anthocyanin levels between ‘Granny Smith’ and ‘Golden Delicious’ can be explained by differential accumulation of *MdMYB1*-specific mRNA. Different levels of *MdMYB1* transcripts in the two cultivars are associated with methylation levels in the promoter region. Hypomethylation of the *MdMYB1* promoter is correlated with the formation of red pigmentation in ‘Granny Smith’ fruit skins. As a result, red pigmentation in Granny Smith’ was more intense than in ‘Golden Delicious’ fruits after bag removal.

**Electronic supplementary material:**

The online version of this article (10.1186/s12870-018-1320-7) contains supplementary material, which is available to authorized users.

## Background

Skin coloration is one of the most important traits related to market value and acceptance in apple (*Malus domestica* Borkh.) fruits, and is ascribed mainly to the presence of anthocyanin pigments. Anthocyanins comprise a major class of flavonoid pigments, and are an excellent source of antioxidants, with nutritional value and potential health benefits [1–3]. The accumulation of anthocyanins is influenced by the intensity and duration of illumination, and anthocyanins are responsible for the red coloration in apple skins [4, 5]. The major anthocyanin pigment present in apple skin is cyanidin 3-galactoside [6].

Anthocyanin biosynthesis is well characterized, and is regulated in vivo by a number of structural and regulatory genes [7, 8]. The structural genes directly involved in the biosynthesis of anthocyanins are chalcone synthase (*CHS*), chalcone isomerase (*CHI*), flavonone 3-hydroxylase (*F3H*), dihydroflavonol 4-reductase (*DFR*), anthocyanidin synthase (*ANS*), and UDP-glycose: flavonoid 3-O-glycosyltransferase (*UFGT*) [9–11]. The regulatory genes encode members of several transcription factor families, and include R2R3 MYB transcription factors, basic helix-loop-helix (bHLH) transcription factors, and WD40 proteins [12, 13]. In apple, *MdTTG1* encodes a WD40 protein [14].

*MdMYB1* is associated with red pigmentation in apple fruit skin [10]. Transcription levels of *MdMYB1* have been shown to correlate with anthocyanin synthesis in red-skinned cultivars and in red regions of the fruit skin [15]. *MdMYB1* has several alleles; *MdMYB1–1* and *MdMYB1–2* share 100% amino acid sequence identity in the coding regions, but there are eight nucleotide differences between the two promoter regions [10]. In addition, *MdMYB10* is another allele of *MdMYB1* that is mainly associated with red pigmentation in fruit flesh and leaves [16, 17]. The *MdMYB1–1* allele co-segregates with red skin phenotypes and regulates anthocyanin synthesis in apple skins under natural conditions. However, the *MdMYB1–2* allele cannot confer red pigmentation in apple skins owing to poor expression levels in non-red-skinned cultivars [10]. Moreover, when dark-grown apple fruits were re-exposed to sunlight, *MdMYB1* expression increased, and the levels were correlated with anthocyanin accumulation in the fruit skin [18–20]. Currently, little is known about how *MdMYB1* expression is regulated in apple fruits after bag removal.

Transposon insertions and deletions in *MYB* promoter regions can lead to skin color changes in apple [17] and grape (*Vitis vinifera*) [21]. DNA methylation is a prominent epigenetic modification in eukaryote genomes, and is another cause of mutation [22, 23] where cytosine methylation is observed at positions with CG, CHG, and CHH sequence contexts (where H is A, C, or T) [24]. Dynamic DNA methylation changes are induced by environmental stress, which were observed in maize (*Zea mays* L.) [24], tobacco (*Nicotiana tabacum)* [25], and pea (*Pisum sativum* L.) [26]. DNA methylation of coding regions generally does not affect gene expression, but methylation in promoter regions inhibits transcription [27, 28]. The binding of transcription factors is inhibited by DNA promoter methylation, and the high degree of methylation in gene promoter regions results in low expression in fruits. Compared with the red-skinned Max Red Bartlett pear (*Pyrus communis*), increased methylation of the *PcMYB* promoter and reduced expression of *PcMYB* were shown in a variety with the green-skin phenotype [29]. In addition, the red stripes of ‘Honeycrisp’ showed lower overall methylation levels than green stripes throughout promoter region of *MdMYB10* [11]. 5-Aza-2′-deoxycytidine (5-aza-dC) is an analogue of cytosine that can irreversibly bind the methyltransferase enzymes when incorporated into DNA; thus, it is a strong inducer of DNA demethylation [30]. Exposure to exogenous 5-aza-dC in plants can induce phenotypic trait variation by restricting DNA methylation [31].

**Fig. 1:**
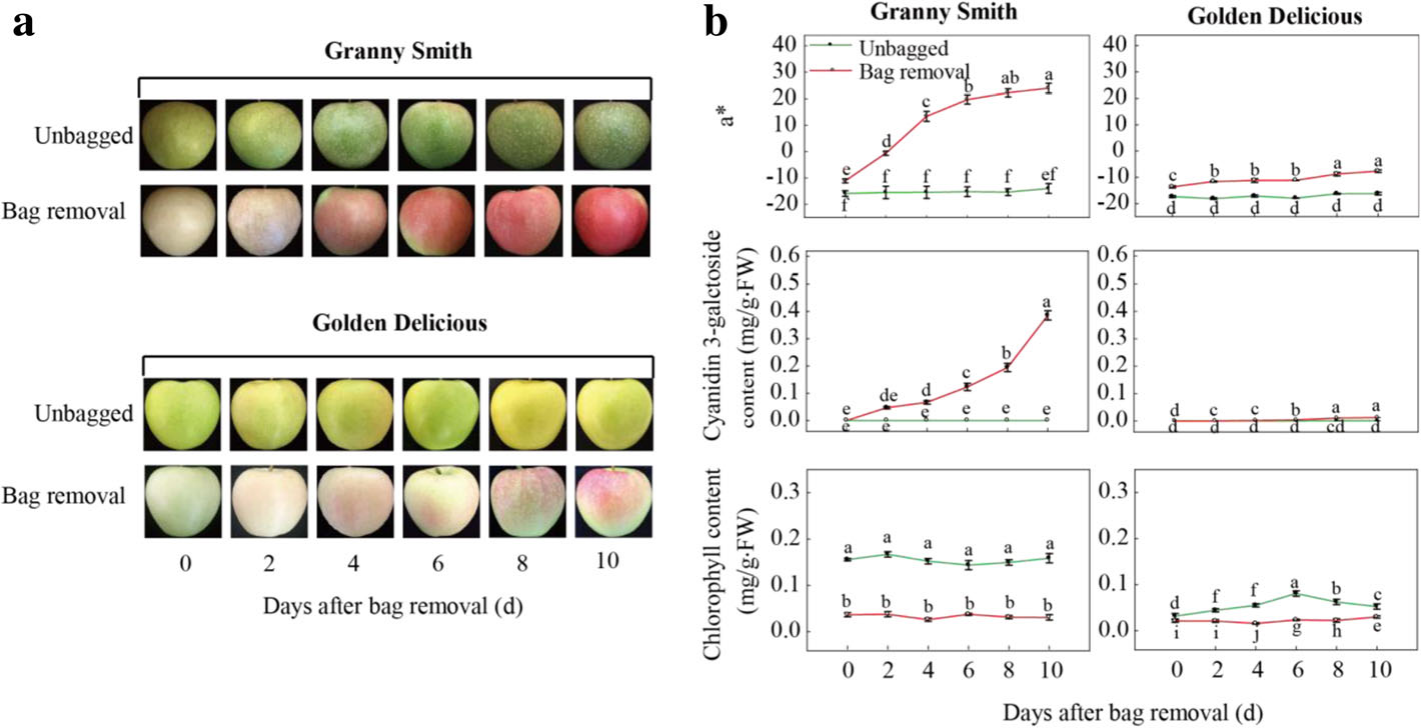
Changes in skin color and pigments in non-red skinned cultivars ‘Granny Smith’ and ‘Golden Delicious’ after bag removal. **a**: Color development in apple skin of the two cultivars 0–10 days after bag removal; **b**: Changes in color parameter a* value, cyanidin3-galactoside content, and chlorophyll content in fruit skin of two apple cultivars after bag removal. Data represent means ± standard deviation of four replicate samples. Different lower case letters indicate significant differences by Tukey’s multiple range test *(p* < 0.05)

‘Granny Smith’ is a green-skinned apple variety that originated in Australia, and the fruits can turn cardinal red after bag treatment during fruit ripening in the Loess Plateau region of China [18, 32], whereas fruits of the yellow-skinned cultivar ‘Golden Delicious’ display a pink blush after bag removal (Fig. 1a). Moreover, ‘Granny Smith’ and ‘Golden Delicious’ are two important commercial cultivars and valuable breeding materials. It is interesting to explain the phenomena of differential pigmentation and valuable for apple breeding programs that take genetic engineering approaches to improve fruit color. Takos et al. (2006) described the two alleles of *MdMYB1*, and showed that the *MdMYB1–1* allele was not present in ‘Delicious’, but was found in ‘Granny Smith’ [10]. However, it is unclear whether methylation is responsible for color differences in the two non-red apple cultivars after bag removal. Therefore, we hypothesized that hypomethylation of the *MdMYB1* promoter was a causative factor for different pigmentation in the two cultivars. In this study, we analyzed the methylation of the *MdMYB1* promoter in non-red skinned cultivars ‘Granny Smith’ and ‘Golden Delicious’, and discuss the potential mechanisms behind the regulation of pigmentation in apple fruit skins.

## Results

### Color development in apple skin after bag removal

Both ‘Granny Smith’ and ‘Golden Delicious’ apples turned red/pink in color after the bagged fruits were exposed to sunlight, and the red pigmentation in the green cultivar ‘Granny Smith’ was more intense than in the yellow cultivar ‘Golden Delicious’ (Fig. 1a). The values of a* for the two cultivars, which are negative for green and positive for red, increased with increasing time after bag removal. The fruits removed from the bags showed higher a* values than those of unbagged fruits. The values of a* in the unbagged fruits remained fairly constant. On the first day after bag removal, the a* value of ‘Granny Smith’ was approximately − 11, and increased to 24.0 at 10 days after bag removal (DABR). In ‘Golden Delicious’, the a* values remained negative during the whole experiment, with only a slight increase after the fruits were removed from the bags (Fig. 1b).

### Changes in anthocyanin and chlorophyll contents during fruit coloration

The contents of anthocyanins and chlorophyll in the skins of the two apple cultivars were measured after bag removal. When the dark-grown fruits were exposed to light, the anthocyanin content increased significantly in ‘Granny Smith’. But when compared with ‘Granny Smith’, only trace amounts of anthocyanin were observed in the skins of ‘Golden Delicious’ fruits. At 10 DABR, the cyanidin 3-galactoside content in the skins of ‘Granny Smith’ fruits removed from the bags was 0.39 mg·g^− 1^, which was approximately 30.9-fold higher than the content in ‘Golden Delicious’ fruit skins. Additionally, the content of chlorophyll in ‘Granny Smith’ was significantly lower in the fruits removed from the bags than it was in unbagged fruits. Nevertheless, chlorophyll contents in ‘Golden Delicious’ fruits removed from the bags remained at ~ 0.01 mg·g^− 1^, and the content in unbagged fruits increased slightly from 2 to 6 DABR (Fig. 1b).

### Expression of anthocyanin biosynthesis genes in apple skin after bag removal

**Fig. 2:**
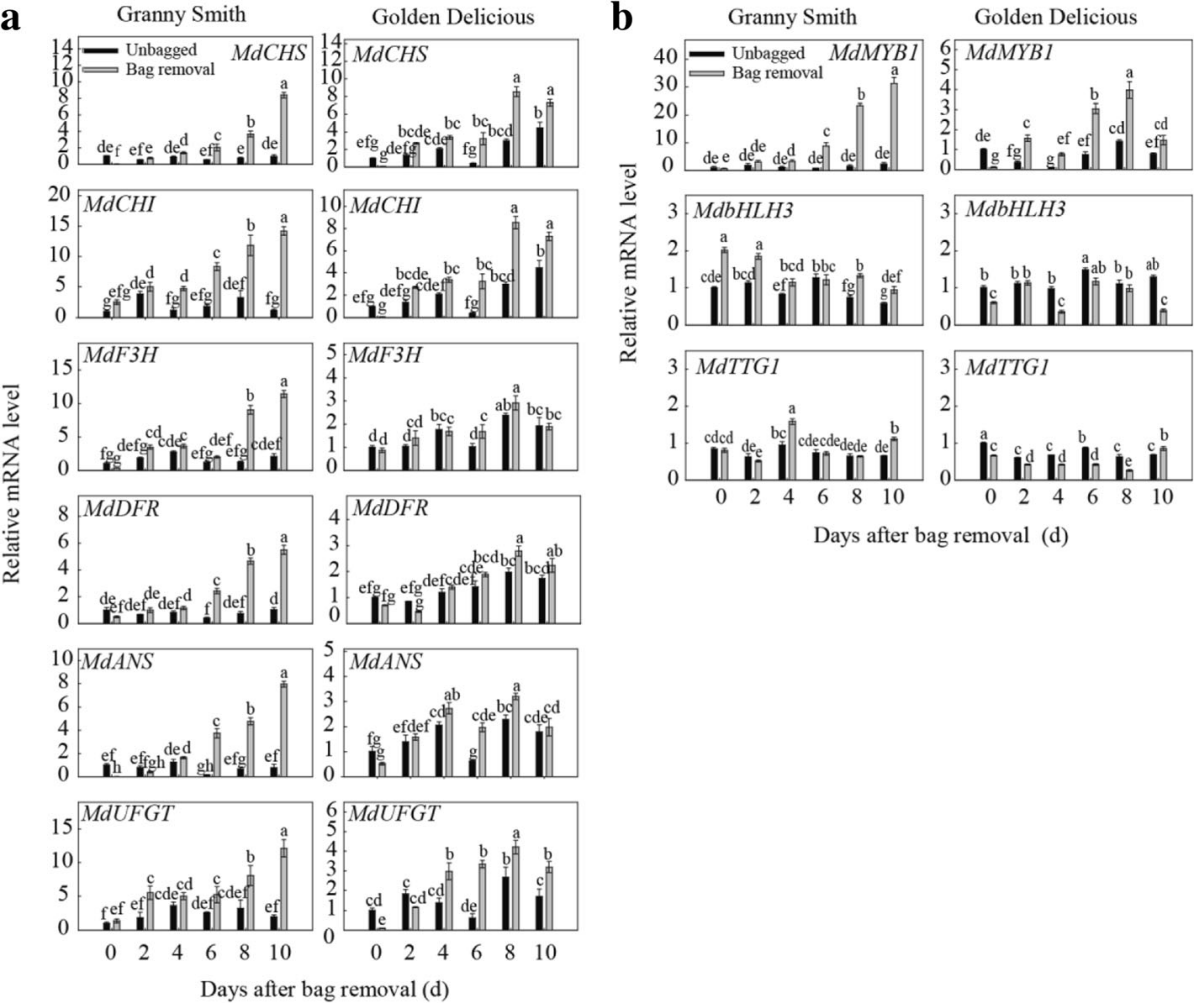
Relative expression levels of genes related to anthocyanin biosynthesis in ‘Granny Smith’ and ‘Golden Delicious’ skins after bag removal. **a**: Expression of structural genes in the two cultivars after bag removal; **b**: Expression of regulatory genes in the two cultivars after bag removal. Data represent means ± standard deviation of four replicate samples. Different lower case letters indicate significant differences by Tukey’s multiple range test (*p <* 0.05)

To determine whether the anthocyanin biosynthetic genes were involved in the trait differences between the fruits removed from the bags and the unbagged fruits, the expression of six structural genes, *MdCHS*, *MdCHI*, *MdF3H*, *MdDFR*, *MdANS*, and *MdUFGT*, and three regulatory genes, *MdMYB1*, *MdbHLH3*, and *MdTTG1*, were quantified by real-time PCR. When bagged fruits were exposed to light, transcript levels of *MdMYB1* and the six structural genes increased immediately in both cultivars, and the expression pattern of the structural genes basically paralleled *MdMYB1* transcription level (Fig. 2a, b). In addition, the transcription levels of structural genes in ‘Golden Delicious’ were lower than those in ‘Granny Smith’ after bag removal (Fig. 2a). *MdMYB1* transcripts remained at a low level in unbagged fruits of ‘Granny Smith’ and ‘Golden Delicious’ (Fig. 2b). The *MdMYB1* transcript levels showed the same trend as anthocyanin content in the two cultivars (Figs. 1b, 2b), and the mRNA levels were higher in fruits removed from the bags compared to the levels in unbagged fruits. The relative mRNA levels of the two transcription factor genes *MdbHLH3* and *MdTTG1* in ‘Granny Smith’ showed only slight increases in the fruits removed from the bags, but the expression patterns did not parallel the anthocyanin levels (Fig. 1b). In ‘Golden Delicious’, there were low transcription levels of *MdbHLH3* and *MdTTG1* after the bagged fruits were exposed to sunlight. In the fruits of ‘Granny Smith’ that were removed from the bags, the *MdMYB1* transcript levels increased with time, and were approximately 13.4-fold higher than the levels in unbagged fruits at 10 DABR. However, *MdMYB1* mRNA levels in ‘Golden Delicious’ fruits removed from the bags reached their highest level at 8 DABR and were 1.88-fold higher than in unbagged fruits (Fig. 2b). These results indicate that *MdMYB1* expression was induced in non-red skin cultivars when the dark-grown apples were exposed to light.

### Differences in the *MdMYB1* promoter sequences between the two apple cultivars

**Fig. 3:**
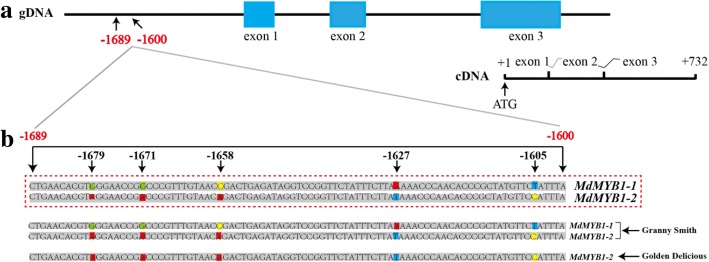
*MdMYB1* genomic DNA in ‘Granny Smith’ and ‘Golden Delicious’. **a**: Diagrammatic representation of genomic organization of the *MdMYB1* gene (not drawn to scale); the numbers refer to positions relative to the first nucleotide of the start codon (ATG); the coding sequences (exons) are shown as blue boxes and noncoding sequence is shown as a black line. Exons, introns, and the promoter region are labeled. **b**: Alignment of the promoter region sequences around − 2000 bp from the translation start codon of the *MdMYB1* gene. The two reference sequences, *MdMYB1–1* (GenBank: DQ886414.1) and *MdMYB1–2* (GenBank: DQ886415.1), are enclosed by a dashed red box. The nucleotide polymorphisms are identified in the 5′ upstream region of the genomic sequence (− 1690 to − 1601 bp) of the two *MdMYB1* alleles

We characterized the *MdMYB1* coding and upstream regions in order to determine whether sequence polymorphisms could possibly explain the different pigmentation patterns. Genomic DNA fragments encompassing approximately 6 kb of *MdMYB1* and including ~ 2 kb of the promoter to the stop codon were isolated from genomic DNA of ‘Granny Smith’ and ‘Golden Delicious’. Five single-nucleotide polymorphisms located in the region between − 1689 bp and − 1600 bp (upstream of the transcription start codon) were used for representative. PCR and DNA sequencing showed that there were no differences between the *MdMYB1* coding regions in ‘Granny Smith’ and ‘Golden Delicious’ (data not shown)*.* Analysis of the *MdMYB1* promoter regions revealed the presence of two *MdMYB1* alleles (*MdMYB1–1* and *MdMYB1–2*) in the green-fruited cultivar ‘Granny Smith’, while only *MdMYB1–2* was present in the yellow-fruited cultivar ‘Golden Delicious’ (Fig. 3b).

### Variation in methylation levels in the *MdMYB1* promoter in apple skins after bag removal

**Fig. 4:**
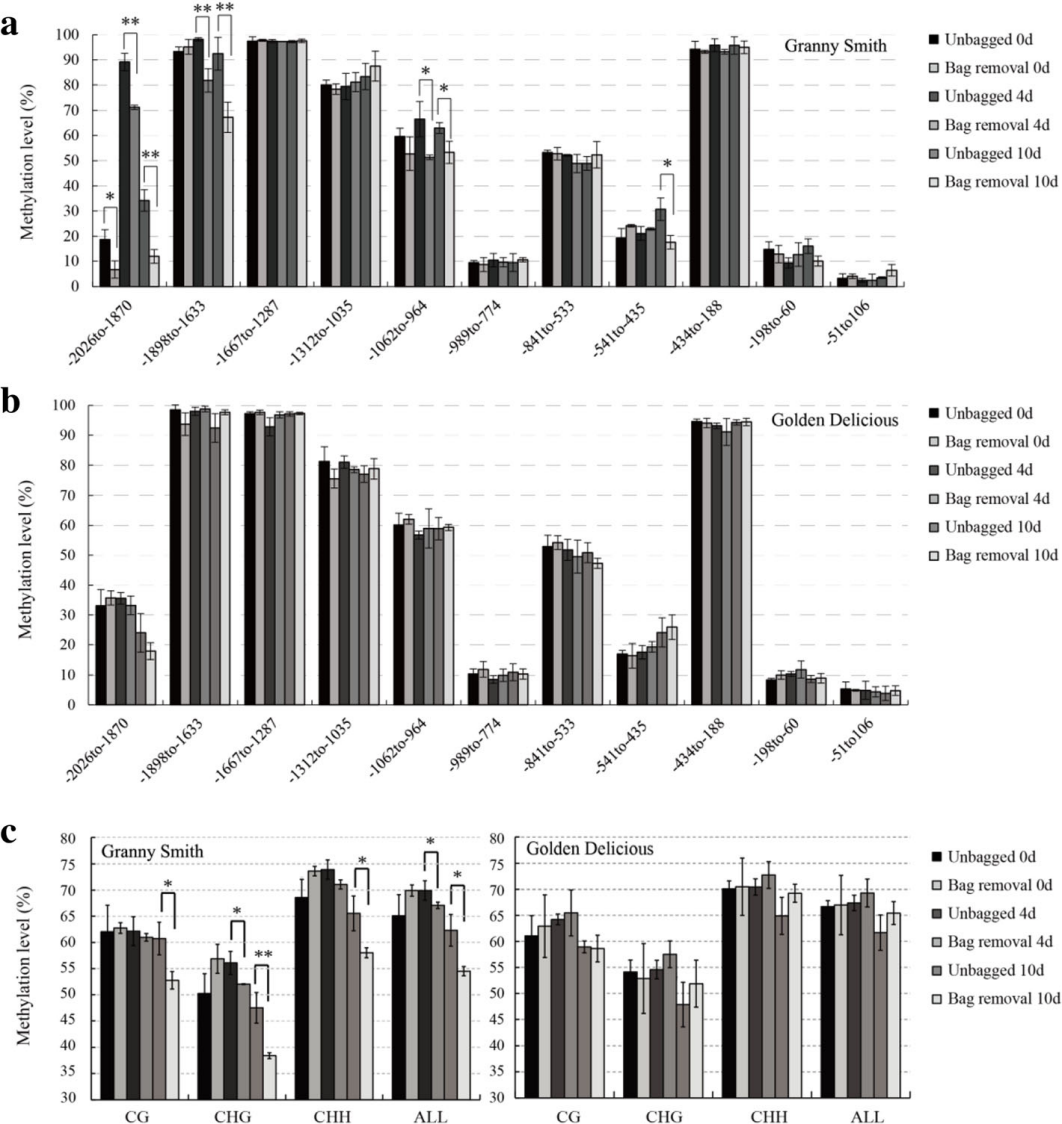
Cytosine methylation levels in the 5′ upstream region of the *MdMYB1* gene in fruit skin after bag removal. Cytosine methylation levels in the *MdMYB1* promoter estimated in ‘Granny Smith’ (**a**) and ‘Golden Delicious’ (**b**). **c**: Methylation levels for the different cytosine contexts in the *MdMYB1* promoter in ‘Granny Smith’ and ‘Golden Delicious’. Fruit skins were harvested at 0, 4, and 10 **d** after bag removal treatment. The product of bisulfite modification was used as a template to amplify the *MdMYB1* promoter region using eleven primer pairs, and twenty independent clones from each PCR were sequenced and analyzed using the Kismeth software. The X axis in A and B indicates the nucleotide positions relative to the ATG translation start site. H indicates A, C, or T. Asterisks indicate significantly different values (* *p <* 0.05; ** *p <* 0.01)

Methylation of the *MdMYB1* promoter regions (conserved sequences were used for both *MdMYB1–1* and *− 2*) in ‘Granny Smith’ and ‘Golden Delicious’ were detected by bisulfite sequencing-PCR. Bagged fruits were sampled at 0, 4, and 10 DABR along with the unbagged samples at the same times. In total, 11 DNA fragments spanning 2 kb of the *MdMYB1* promoter region were evaluated. Overall, in both cultivars, four 5′ upstream regions − 1898 to − 1633 bp, − 1667 to − 1287 bp, − 1312 to − 1035 bp, and − 434 to − 188 bp, showed high methylation levels (~ 80–90%); methylation levels of the regions from − 1062 to − 964 bp and − 841 to − 533 bp were ~ 60%, whereas the methylation levels of the regions from − 989 to − 774 bp, − 541 to − 435 bp, − 198 to − 60 bp, and − 51 to 106 bp were < 30%. A similar pattern of *MdMYB1* methylation was observed in ‘Granny Smith’ and ‘Golden Delicious’. In addition, ‘Granny Smith’ had low methylation levels (5–20%) in the − 2026 to − 1870 bp promoter region at 0 DABR, which reached the highest methylation levels (70–90%) at 4 DABR, and returned to lower levels (10–30%) at 10 DABR. However, the methylation levels in the − 2026 to − 1870 bp fragment remained at 20–40% in ‘Golden Delicious’ skins (Fig. 4a and b).

Significant differences in methylation levels were observed in some regions of the ‘Granny Smith’ *MdMYB1* promoter; − 2026 to − 1870 bp, − 1898 to − 1633 bp, − 1062 to − 964 bp, and − 541 to − 435 bp. In the skin of ‘Granny Smith’ fruits removed from the bags, the methylation level from − 541 to − 435 bp was significantly lower than it was in unbagged fruits at 10 DABR. At 4 DABR, the methylation level in the region from − 1062 to − 964 bp was 15.08% lower in fruits removed the bags than in unbagged fruits, and remained low at 10 DABR. Compared with the unbagged fruits, the methylation level in the − 1898 to − 1633 bp region in fruits removed from the bags at 4 and 10 DABR decreased to 10.91 and 24.58%, respectively. Moreover, the methylation level from − 2026 to − 1870 bp was significantly lower in fruits removed from the bags than in unbagged fruits at 0, 4, and 10 DABR (Fig. 4a). However, methylation levels in the 11 DNA fragments in the ‘Golden Delicious’ *MdMYB1* promoter region showed no significant differences between the unbagged fruits and those removed from the bags (Fig. 4b).

The complete sequences of the *MdMYB1* promoter region were assembled from the 11 upstream DNA fragments after bisulfite conversion. The results indicate that cytosine methylation in the promoter region of the *MdMYB1* gene is found in all three methylation contexts: CG, CHG, and CHH (where H is A, C or T). In ‘Granny Smith’, the methylation level of CHG-type cytosines was significantly lower in the fruit removed from the bags than in the unbagged fruit at 4 DABR. In addition, compared with the unbagged fruit, the CG, CHG, and CHH cytosine methylation in fruits removed from the bags decreased significantly to 7.98, 9.08, and 7.54% at 10 DABR. Furthermore, methylation levels throughout the *MdMYB1* promoter region in ‘Granny Smith’ were significantly lower in fruits removed from the bags than the levels in unbagged fruits at 4 and 10 DABR, but methylation levels in ‘Golden Delicious’ were not significantly different between the two treatment groups (Fig. 4c). Similarly, the cytosine methylation levels of the CG, CHG, and CHH were not significantly different between the unbagged fruits and those removed from the bags in ‘Golden Delicious’, which is consistent with our preliminary results (Fig. 4b).

### Changes in anthocyanin accumulation after 5-aza-dC treatment

**Fig. 5:**
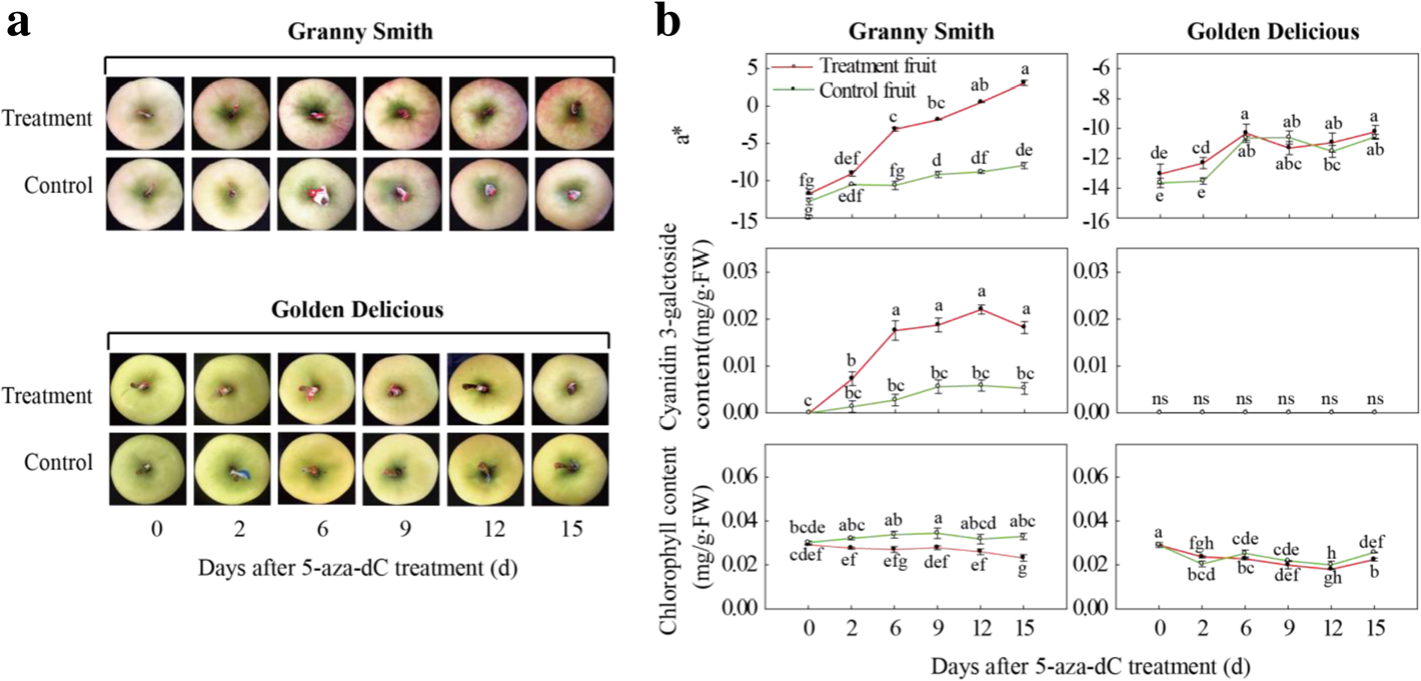
Effect of 5-aza-2′-deoxycytidine (5-aza-dC) treatment on pigments in ‘Granny Smith’ and ‘Golden Delicious’ fruit skins after bag removal. **a**: Color changes in apple skin of the two cultivars after 5-aza-dC treatment. **b**: Changes in color parameter a*, cyanidin3-galactoside content, and chlorophyll content in fruit skin of two apple cultivars after 5-aza-dC treatment. Data represent means ± standard deviation of four replicate samples. Different lower case letters indicate significant differences by Tukey’s multiple range test (*p* < 0.05), and ns indicates not significant

In order to further explore the relationship between hypomethylation and the red skin coloration, the fruits removed from the bags were treated with 5-aza-dC. Compared with control fruits, ‘Granny Smith’ gradually and markedly turned red in color during the 5-aza-dC treatment, whereas ‘Golden Delicious’ fruits did not change color (Fig. 5a). When the 5-aza-dC-treated apples were exposed to light, the a* values and anthocyanin contents in the skins of ‘Granny Smith’ fruits increased, and were significantly higher in the treated fruits than in the control fruits. However, in ‘Golden Delicious’, the a* value remained negative and no anthocyanins were detected in either of the two groups during the time-course of 5-aza-dC treatment. The chlorophyll contents were significantly reduced in the treated fruits of ‘Granny Smith’, but there was slight difference between the treated and control fruits in ‘Golden Delicious’ (Fig. 5b).

**Fig. 6:**
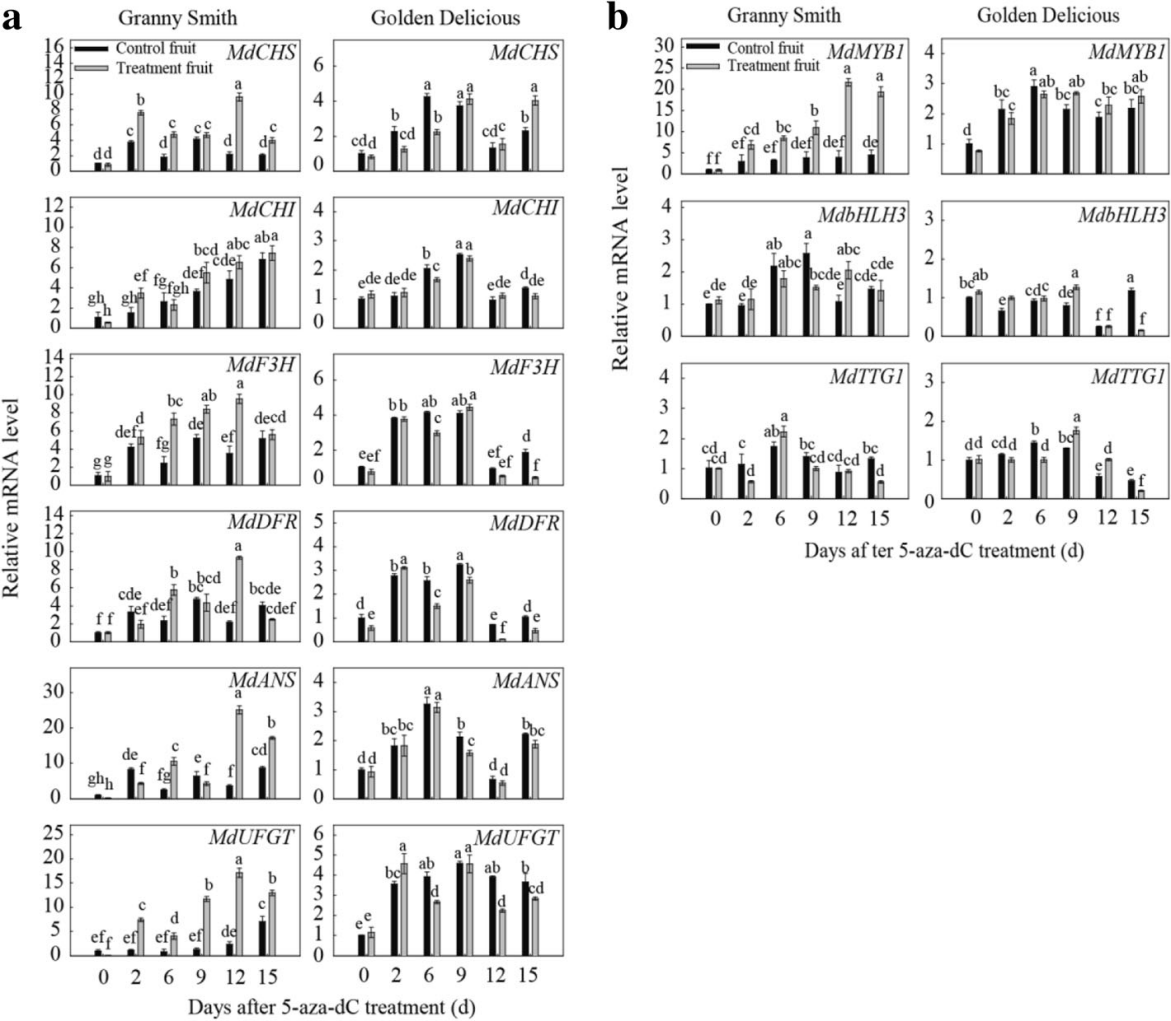
Relative expression levels of genes related to anthocyanin biosynthesis in skins of ‘Granny Smith’ and ‘Golden Delicious’ fruits after 5-aza-2'-deoxycytidine (5-aza-2’-dC) treatment. **a**: Structural gene expression in the two cultivars; **b**: Regulatory gene expression in the two cultivars. Data represent means ± standard deviation of four replicate samples. Different lower case letters indicate significant differences by Tukey’s multiple range test (*p <* 0.05)

The overall trends of transcription of *MdMYB1* and the six structural genes *MdCHS*, *MdCHI*, *MdF3H*, *MdDFR*, *MdANS*, and *MdUFGT* in ‘Granny Smith’ fruit skins were up-regulated, and the expression of *MdCHI*, *MdDFR* and *MdANS* showed slightly fluctuations during the period of 5-aza-dC treatment (Fig. a, b). Moreover, expression of the structural genes followed a trend similar to that seen for *MdMYB1*. The *MdMYB1*-specific transcripts in the treated ‘Granny Smith’ fruits reached their highest levels at 12 days after exposure of 5-aza-dC-treated fruits to light (DAFT), approximately 4.31-fold higher than in control fruits, and then remained at a high level, which correlated with the anthocyanin content (Fig. 6b). However, transcription of *MdbHLH3* and *MdTTG1* showed only 2- to 3-fold fluctuations between the two treatment groups in the two treated cultivars, but were not correlated with anthocyanin content. Furthermore, transcription of all these genes in ‘Golden Delicious’ showed some fluctuations during the 5-aza-dC treatment, but the fluctuation was less evident between the treatment and control fruits than it was in ‘Granny Smith’ (Fig. 6a, b). These results showed that 5-aza-dC treatment increased the sensitivity of *MdMYB1* to positively regulate anthocyanin synthesis in the skins of ‘Granny Smith’ fruits.

### DNA methylation in the 5’ upstream region of *MdMYB1* after 5-aza-dC treatment

**Fig. 7:**
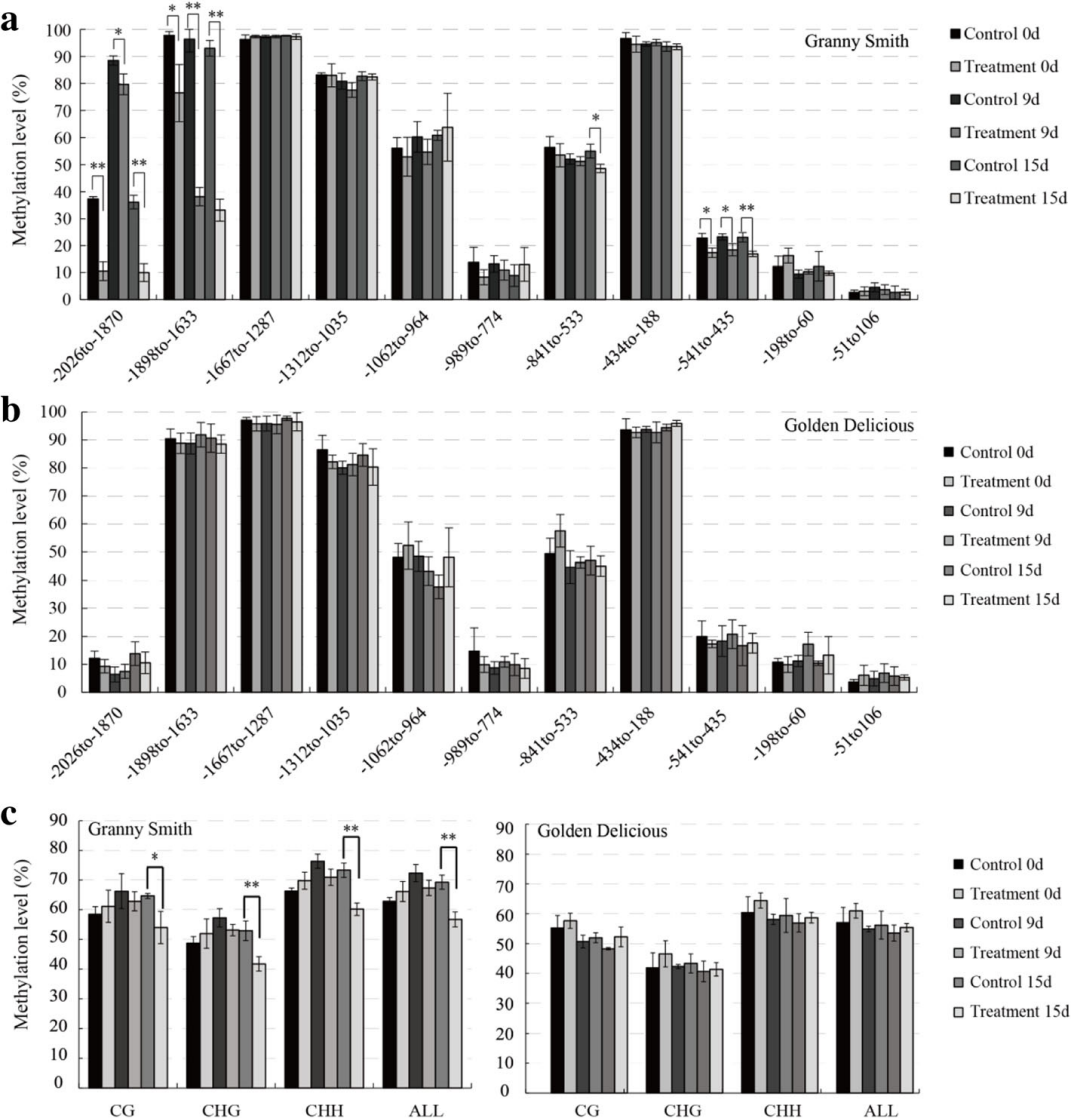
Cytosine methylation levels in the 5′ upstream region of the *MdMYB1* gene in apple skin after 5-aza-2′-deoxycytidine (5-aza-2’-dC) treatment. Cytosine methylation levels of the *MdMYB1* promoter estimated in ‘Granny Smith’ (**a**) and ‘Golden Delicious’ (**b**). **c**: Methylation levels of the three cytosine contexts in the *MdMYB1* promoter in ‘Granny Smith’ and ‘Golden Delicious’. Fruit skins were harvested at 0, 9, and 15 d after 5-aza-dC treatment. The product of bisulfite modification was used as a template to amplify the *MdMYB1* promoter region using eleven primer pairs, and twenty independent clones from each PCR were sequenced and analyzed by Kismeth. The X axis in A and B indicates the nucleotide positions relative to the ATG translation start site. H indicates A, C, or T. Asterisks indicate significantly different values (*p < 0.05; **p < 0.01)

The methylation levels of the 11 *MdMYB1* promoter region DNA fragments from peel samples of ‘Granny Smith’ and ‘Golden Delicious’ were measured at 0, 9, and 15, DAFT (Fig. 7a, b). The methylation levels of three fragments: − 2026 to − 1870 bp, − 1898 to − 1633 bp, and − 541 to − 435 bp in ‘Granny Smith’ showed significant differences between the treated apples and the controls (Fig. 7a), which corresponded with the results from the bag removal treatment (Fig. 4a). Compared with the ‘Granny Smith’ control fruits, the methylation levels of the three regions in treated fruits decreased significantly at 0, 9, and 15 DAFT. Furthermore, the region from − 841 to − 533 bp in ‘Granny Smith’ showed a significantly lower level of methylation in treated fruits than in control fruits at 15 DAFT (Fig. 7a). The methylation levels of CG, CHG, and CHH cytosines in treated fruits of ‘Granny Smith’ were 10.7, 11.17, and 13.14% lower, respectively, than those in control fruits at 15 DAFT. In addition, methylation levels throughout the *MdMYB1* promoter region in ‘Granny Smith’ decreased significantly in the treatment groups at 15 DAFT (Fig. 7c). However, in ‘Golden Delicious’, there were negligible differences in methylation levels between the treatment and control groups (Fig. 7b, c), which corresponded with the results from the bag treatment (Fig. 4b, c).

## Discussion

### Up-regulation of *MdMYB1* transcription is associated with anthocyanin accumulation in skins of non-red apple cultivars

After removing the fruits from the bags, the contents of chlorophyll and anthocyanins in the apple skins were significantly decreased and increased, respectively, compared with unbagged fruits (Fig. 1b). This suggests that reduction in chlorophyll content may be more conducive to the accumulation of anthocyanins, but is not the main reason for the red skin coloration in fruits removed from the bags; the enhanced red pigmentation can be attributed to anthocyanin content. Expression of the structural genes in the anthocyanin biosynthesis pathway was significantly increased in the fruit skins of two apple cultivars after bag removal (Fig. 2a). It has been considered that sunlight exposure promotes anthocyanin synthesis in non-red skin cultivars after bag removal [18].

Transcription factors such as *MYB1* are important regulators in the biosynthesis of anthocyanins in apple [17]. In the present study, the expression of the *MdMYB1* gene showed a strong correlation with the changing patterns of anthocyanin accumulation after bag removal and 5-aza-dC treatment, also suggesting its important role in this process. In the fruits of ‘Granny Smith’ that were removed from the bags, the *MdMYB1* mRNA levels increased markedly at 10 DABR compared to the controls. In contrast, *MdMYB1* transcripts in ‘Golden Delicious’ fruits that had been removed from the bags only slightly fluctuated during the bag removal treatment (Fig. 2b). To ascertain the reason for the differences in *MdMYB1* expression, we focused on the function of *MdMYB1* in anthocyanin biosynthesis in ‘Granny Smith’ and ‘Golden Delicious’ fruit skins. Comparing the coding region sequences of *MdMYB1* revealed no differences between the two cultivars (Fig. 3), suggesting that other factors upstream of the gene may contribute to the expression differences between ‘Granny Smith’ and ‘Golden Delicious’.

The *MdMYB1–1* allele co-segregates with red skin phenotypes [10], but the skin of ‘Granny Smith’ is green under natural conditions, though the genome of this cultivar harbors the *MdMYB1–1* allele. This indicates that the presence of *MdMYB1–1* probably does not explain the formation of red skins in ‘Granny Smith’ fruits after bag removal. It is generally considered that *MdMYB1–2* alleles cannot confer red pigmentation in apple skins under natural conditions [10]. In this respect, we found that both ‘Granny Smith’ and ‘Golden Delicious’ carry the *MdMYB1–2* allele. These results suggest that the differences in skin anthocyanin biosynthesis between ‘Granny Smith’ and ‘Golden Delicious’ could result from methylation of the *MdMYB1* promoter region.

### Methylation of the *MdMYB1* promoter affects anthocyanin accumulation after bag removal and 5-aza-dC treatment

DNA methylation changes the regulation of transcription and can alter the magnitude of gene expression [31]. In the present study, methylation analysis of the *MdMYB1* promoter showed that methylation levels were inversely correlated with *MdMYB1* expression in the skins of ‘Granny Smith’ apples. Hypermethylation was associated with low anthocyanin levels in unbagged and control (without 5-aza-dC treatment) fruits, while a low level of methylation was associated with high anthocyanin content in fruits removed from the bags and in 5-aza-dC treated fruits. The cytosine methylation levels in the − 2026 to − 1870 bp promoter fragment varied during the time of bag removal treatment in ‘Granny Smith’, reaching the highest levels at 4 DABR, and decreased at 10 DABR. Similar results were also observed in the 5-aza-dC treatment; this suggests that methylation in the *MdMYB1* region promoter may also be developmentally controlled. It would be interesting to investigate the methylation behavior in apple coloring to further illustrate the epigenetic role of methylation in regulation of *MdMYB1*. In addition, the context of DNA methylation in color variation in maize is largely due to methylation of CG/CHG cytosines [33]. However, the methylation levels of CHH, CHG, and CG cytosine contexts all decreased in fruits that had been removed from the bags and in 5-aza-dC treated fruits. This indicates that the regulatory mechanisms controlling DNA methylation could be more complicated in apple than in maize, and the existence of three types of cytosine methylation in the *MdMYB1* promoter might increase the impact on red pigmentation in ‘Granny Smith’ fruits.

The differential methylation found in regions − 2026 to − 1870 bp, − 1898 to − 1633 bp, and − 541 to − 435 bp of the *MdMYB1* promoter region between the control and 5-aza-dC-treated groups corresponded with the results from the bag removal treatment, indicating that the three regions could play essential roles in red pigmentation of ‘Granny Smith’ fruit skin. The results were similar to the DNA methylation patterns of ‘Honeycrisp’ and ‘Royal Gala’; compared with green-striped apple, red-striped apple showed lower methylation levels in the regions from − 2026 to − 1870 bp and − 1898 to − 1633 bp [11]. The expression of *MYB* genes is affected by several environmental stimulators, such as light, low temperature, and dehydration stress, which have been well characterized [10, 33, 34]. *MYB* transcription factors also act in biotic stress responses, such as those conditioned by abscisic acid, gibberellin, and jasmonate [35–38]. Moreover, the transcription of *MYB* genes can be activated by MYB proteins through auto-regulatory mechanisms in the promoter [17]. In our work, the highly differentially methylated regions of the *MdMYB1* promoter (− 2026 to − 1870 bp, − 1898 to − 1633 bp, and − 541 to − 435 bp) comprise numerous *cis*-acting elements, including *MYB*-binding sites, abscisic acid-, salicylic acid-, and gibberellin-responsive elements, light-responsive elements, and dehydration-responsive elements (Additional file 1: Table S1). This suggests that methylation within the three promoter regions may reduce not only the binding of MYB proteins, but also the impact of the environmental stimulators and biotic stress responses. Demethylation in the − 2026 to − 1870 bp, − 1898 to − 1633 bp, and − 541 to − 435 bp regions of the *MdMYB1* promoter might be an important mechanism regulating the expression of *MdMYB1*, resulting in anthocyanin accumulation in the skins of ‘Granny Smith’ fruits.

### The molecular mechanism of red/pink pigmentation in ‘Granny smith’ and ‘Golden delicious’

**Fig. 8:**
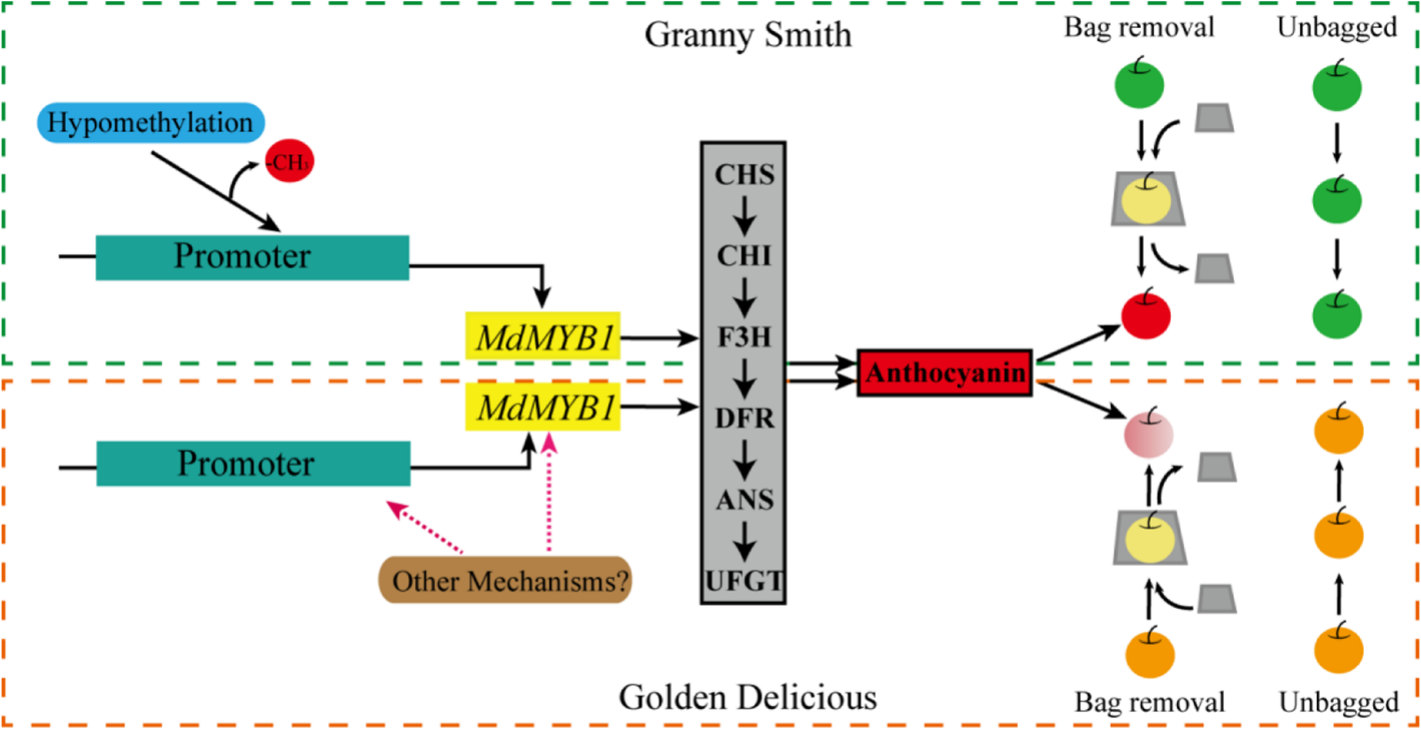
Model showing the proposed molecular mechanisms underlying red/pink pigmentation in ‘Granny Smith’ and ‘Golden Delicious’ apple skins. Hypomethylation of the *MdMYB1* promoter region increased the expression of *MdMYB*, which regulates expression of the structural genes involved in anthocyanin biosynthesis. As a result, the content of anthocyanins is greatly increased, causing the formation of red pigmentation in skins of ‘Granny Smith’ fruits. There might be other mechanisms responsible for the pink coloration of ‘Golden Delicious’ skins. Red pigmentation of ‘Granny Smith’ skins was more intense than in ‘Golden Delicious’ after bag removal. The illustration shows the treatments used in this study. Fruits of Green-skinned cultivar ‘Granny Smith’ and yellow-skinned cultivar ‘Golden Delicious’ were white in color when they were bagged, and developed red and pink pigments, respectively, after bag removal. The dashed green and red border lines indicate the mechanism of formation of pigmentation in skins of ‘Granny Smith’ and ‘Golden Delicious’, respectively. The red ball indicates -CH_3_ (methyl group)

Bagging is an effective treatment to enhance the red pigmentation of fruit skins [18, 39, 40]. Unlike the bright red color induced in green-skinned ‘Granny Smith’, yellow-skinned ‘Golden Delicious’ apples appeared pale-pink after bag removal. The different methylation patterns in the *MdMYB1* promoter in the two cultivars raises the question of how the different pigmentation levels and patterns form after bag removal. In ‘Granny Smith’, the expression of *MdMYB1* was increased by hypomethylation of its promoter after bag removal. As a result, the expression of six structural genes, which are involved in anthocyanin biosynthesis and are regulated by *MdMYB1*, also increased. This would be expected to be associated with the increase in anthocyanin biosynthesis and the formation of red skins in ‘Granny Smith’ fruits (Fig. 8). However, in ‘Golden Delicious’, there were negligible differences in *MdMYB1* methylation levels between unbagged fruits and those removed from the bags, which corresponded to the results from the 5-aza-dC treatment. We speculated that during apple fruit development, differences in *MdMYB1* methylation are present among individual cells, as previously indicated by Cocciolone and Cone [41]. Telias et al. [11] indicated that the variable methylation in the *MYB10* promoter gives rise to variable color patterns in the skin of apple. Similar results were found in maize, where *Ufo1* controls methylation levels in *p1* (an *MYB* transcription factor), and *Ufo1* activity may also produce variegation in the maize pericarp [33]. Furthermore, variation among individuals in the degree of gene methylation has been reported to affect gene expression [42]. Our present work shows that the differences in pigmentation between the two cultivars are a result of the different methylation levels in the *MdMYB1* gene promoter. However, we hypothesize that there are other mechanisms responsible for the pigmentation of ‘Golden Delicious’ fruit skins (Fig. 8), which could be revealed by further studies.

## Conclusions

Differences in anthocyanin accumulation between ‘Granny Smith’ and ‘Golden Delicious’ fruits can be explained by the differential expression of *MdMYB1*. The increase in *MdMYB1*-specific mRNA levels in ‘Granny Smith’ skins is associated with hypomethylation in the promoter region, especially the regions − 2026 to − 1870 bp, − 1898 to − 1633 bp, and − 541 to − 435 bp. Unlike ‘Granny Smith’, ‘Golden Delicious’ fruits did not respond to 5-aza-dC treatment. Hypomethylation of the *MdMYB1* promoter is probably the causative factor in the formation of red pigmentation in ‘Granny Smith’. We suggest that differential methylation of the *MdMYB1* promoter in the two cultivars alters the accumulation of *MdMYB1*-specific transcripts, which in turn affects anthocyanin accumulation. As a result, the red pigmentation induced in Granny Smith’ fruits was more intense than in ‘Golden Delicious’ after bag removal. We hypothesize that there is not just a single mechanism responsible for the coloration pattern, and the pigmentation in ‘Golden Delicious’ fruits warrants further study. However, our findings provide a possible explanation for the variation in bagging-induced pigmentation in non-red skinned apples, and also offer new insights into the regulation of differential skin coloration that results from DNA methylation.

## Methods

### Plant materials and treatment

Fruits of two apple cultivars (at least 192 fruits each cultivar), ‘Granny Smith’ and ‘Golden Delicious’, were collected at the Baishui Apple Experimental Station of Northwest A&F University in Shaanxi, China (35°21′ N, 109°55′ E, elevation 850 m). The two apple cultivars were grafted onto M26 rootstock (*M. domestica* Borkh.) and planted in 4 × 2 m plots. Young fruits were wrapped with two-layer paper bags (inner red, outer brown; Hongtai, Shaanxi, China), at 40 days after full bloom (DAFB). In order to protect bagged fruits from sunburn, the outer papers were removed two days before the inner papers. The ‘Golden Delicious’ outer papers were removed at 120 DAFB, while the ‘Granny Smith’ papers were removed at 160 DAFB as described by Liu et al. (2013) [45]. Fruits without the paper-bagging treatment (unbagged fruits) were used as controls for comparison. The fruits samples were randomly taken from the south side of tree canopy between 8 a.m. and 10 a.m. at 0, 2, 4, 6, 8, and 10 days after the inner papers were removed. On each sampling date, four biological replicates were harvested, with at least four ftuits collected from two trees per replicate. The skin color of fruits was immediately analyzed. Fruit peel (~ 1 mm in thickness) was collected with an apple peeler according to the method described by Qu et al. [43] and fruits from the two trees were pooled as one sample. For cloning *MdMYB1* genomic sequences, young leaves of two apple cultivars were collected on April 26th. Leaves from two trees were pooled as one sample (four samples per cultivar). All samples were immediately frozen in liquid nitrogen and stored at − 80 °C.

For 5-aza-dC treatment, bagged fruits (both inner and outer papers) were removed simultaneously at harvest day. ‘Golden Delicious’ was harvested at 120 DAFB, while ‘Granny Smith’ fruits were harvested at 160 DAFB. At least 144 bagged fruits per cultivar were used, and all fruits were divided into two groups. Four replicates were conducted in each group, with at least 18 fruits per replicate. The first group was coated with 1 mM 5-aza-dC (Sigma, St. Louis, MO, USA) plus 0.1% (*v*/v) Tween-20—this content had been determined in a previous trial (unpublished data) to be effective in apple coloring, and the second (control) group was mock-treated with an equal volume of sterile distilled water. All the treated fruits were placed in one dark chamber (25 °C) for 24 h and were then exposed to white light (540 μmol·m^− 2^·s^− 1^) at 25 °C with a 16 h photoperiod. The fruit samples were treated with 5-aza-dC or sterile distilled water before dark treatment every day until the entire experiment was completed. Apple peels were carefully harvested at six time points (0, 2, 6, 9, 12, and 15 d after the first 24 h dark treatment). On each sampling date, four replicates were taken, with at least three fruits per replicate. The fruits were immediately used for the measurements of skin color, and then the fruit peel (∼1 mm in thickness) was collected with an apple peeler according to the method described by Qu et al. [43]. Fruits from the one replicate were pooled as one sample before freezing in liquid nitrogen. All samples were stored at − 80 °C until use.

### Measurements of skin color, anthocyanin and chlorophyll contents

Skin color was measured using a colorimeter (CR-400, Minolta, Japan). Fruit chromaticity was recorded using the Commission Internationale D’eclairage parameters L*, a*, and b* [5, 44]. Here, we focused on skin color using values of a*. The a* scale extends from − 60 to 60, and is negative for green and positive for red. Measurements were performed on at least 32 fruits (16 for bag removal treatment and 16 for unbagged treatment) or 24 fruits (12 for 5-aza-dC treatment and 12 for control) on each sampling date. For each fruit, color measurements were taken and averaged for five points around the fruit’s equator.

Measurement of anthocyanin content was performed according to the method of Liu et al. [45]. Apple peels (0. 5 g) were finely ground in 5 mL 1% (*v*/v) HCl-methanol for 24 h at 4 °C in the dark. A total of 48 samples from four independent biological replications per cultivar were taken for each experiment. Samples were clarified by centrifugation at 13,000×g for 10 min at 4 °C. The 1.5 mL supernatant was transferred to autosampler vials for high-performance liquid chromatography analysis. Analysis was conducted on a high-performance liquid chromatography with photodiode array detection (Waters, Milford, MA, USA). Anthocyanins were separated on a C18 column (5 μm, 250 × 4.6 mm internal diameter, Waters). Mobile phases were 10% (v/v) formic acid (A) and methanol (B). The gradient conditions were: 0 min, 17%; 1 min, 17%; 15 min, 35%; 20 min, 37%; and 25 min, 100%. The gradient was run at a flow rate of 1 mL min^− 1^ at a column temperature of 40 °C; 10 μL samples were injected; detection wavelength was 520 nm; and post-run time was 15 min. The standard was cyanidin 3-galactoside (Sigma).

Measurement of total chlorophyll was according to the protocol of Lichtenhaler and Wellburn [46], with slight modifications. Briefly, frozen ground peel tissue (0.5 g) was homogenized in 5 mL of 80% acetone. The homogenate was left 24 h in dark conditions and then centrifuged for 20 min at 13,000×g. Absorbance of the supernatant was measured at 663 and 645 nm using a UV-2550 ultraviolet spectrophotometer (Shimadzu Corp., Kyoto, Japan). The chlorophyll concentration was calculated using the equation Ct = 20.2A_645_ + 8.02A_663_. In all, four independent biological replications were performed for each experiment.

Data were expressed as means ± standard deviation. The data were assessed via one-way analysis of variance (one-way ANOVA), followed by Tukey’s test (*P* < 0.05) using SPSS 16.0 Statistics (SPSS Inc., Chicago, IL, USA).

### Real-time quantitative PCR analysis

One microgram of total RNA from each pool was isolated using the Trizol RNA Plant Plus reagent (Tiangen, Beijing, China), which includes RNase-free DNase treatment. Total RNA was adjusted to the same concentration for first-strand cDNA synthesis using a PrimeScript Master Mix Kit (TaKaRa, Dalian, China) according to the manufacturer’s instructions. The six structural genes, *MdCHS*, *MdCHI*, *MdF3H*, *MdDFR*, *MdANS*, and *MdUFGT*, and three regulatory genes, *MdMYB1*, *MdbHLH3*, and *MdTTG1*, were amplified by real-time PCR. Specific PCR primer information is given in Table 1. Real-time PCR amplification and analysis was performed on an iQ5.0 instrument (Bio-Rad, Hercules, CA, USA) using the SYBR Premix Ex Taq Kit (TaKaRa) according to the manufacturer’s instructions. The *Malus domestica* actin gene (GenBank accession number GQ339778.1) was used for normalization of gene expression levels. Data was analyzed by the 2^-∆∆CT^ method [47]. All qRT-PCR experiments were repeated four times, based on four separate RNA extracts from four samples. Differences between the means were analysed using one-way ANOVA followed by post hoc Tukey’s test (*P* < 0.05). A *p*-value of < 0.05 indicated a significant difference.

**Table 1:** DNA sequences of oligonucleotide primers used in real-time PCR and *MdMYB1* genomic sequence analyses

Gene identifier (Genbank)	Gene Name	Sense Primer (5′-3′)	Anti-sense Primer (5′-3′)
AB074485.1	*MdCHS*	AGTGACACCCACCTTGATAG	CTGTCGGGGAGAATGGTTTG
AF494398.1	*MdCHI*	GTTACAGGTCCGTTTGAGAA	AACTTTTCAATGGCTTTGCCTTCTG
AB074486.1	*MdF3H*	CTGCTACTACGCTGACATCC	AATACCCCAGTCCTCACAAG
AF117268.1	*MdDFR*	ATTTATCTTTACGAGCATCC	CCCTATCTCCCTCAACTTCT
AB074487.1	*MdANS*	GTTCCAAATTCCATCGTCAT	TCACCTTTTCCTTGTTCACC
AF117267.1	*MdUFGT*	TCGTAGCCTTCCCTTTCACT	TTATCAATGCTGTTGTTGGAAAAGA
DQ886414.1	*MdMYB1*	GTCGTCGTCAACAAAGAATGG	GGTCCGTGCTAAAGGAGAAT
HM122458.1	*MdbHLH3*	GTAAAGAGTTGCGAAGTGGG	GTAAAGGTTCTGGCTGAGGT
GU173813.1	*MdTTG1*	AGAGCGGAGAACTCGGTGAC	CGATTCGGTGGTGCTGGTG
GQ339778.1	*MdACTIN*	CTGAACCCAAAGGCTAATCG	ACTGGCGTAGAGGGAAAGAA
DQ886414.1/DQ886415.1	PMBY1	AACAGTATCAGTTCGTGCATCAGTG	CTCAACGCACTGCCTGAGAAGATTG
DQ886414.1/DQ886415.1	PMBY2	TGAGTGTGAGAAAAGGTGCCTGGAC	CACAATCTTAAAGGTTTGCTCAGTCGA
DQ886414.1/DQ886415.1	PMBY3	GGATTCAAATAAAGTATCAAACAGG	CAAGCAAGGAAAATAATAGAACACA

### Cloning of the *MdMYB1* genomic sequences

Leaf tissues (0.1 g) were ground in liquid nitrogen. To eliminate residual carbohydrates and polyphenols, interfering solution [0.1 mol·L^− 1^ Tris-HCL (pH 8), 0.02 mol·L^− 1^ ethylene diaminetetraacetic acid (pH 8), 5% (*v*/v) glycerol, 10% (v/v) polyethylene glycol 8000, 2% (v/v) polyvinyl pyrrolidone, and 5% (v/v) β-mercaptoethanol] was added to eight samples (four samples per cultivar), the slurry was incubated on ice for 10 min, and the solution was clarified by centrifugation at 13,000×g for 10 min at 4 °C. After the interfering solution was used three times, the DNA was isolated by the Haymes method [48], or with the DNeasy Plant mini Kit (Qiagen, Valencia, CA, USA).

Primers for amplfication of the *MdMYB1* genomic sequence are given in Table 1. Fragments were amplified using high-fidelity DNA polymerase KOD plus Neo (Toyobo, Tokyo, Japan), using the reaction conditions recommended by the manufacturer. The PCR products were purified after agarose gel electrophoresis using the Wizard SV DNA purification system (Promega, Madison, WI, USA), and cloned into the PMD 18-T vector (TaKaRa). Sequences of 10 independent clones of each fragment per sample were sequenced directly. Promoter sequence analysis was performed using the PLACE Signal Scan Search online database [19].

### Methylation analysis

Bisulfite sequencing-PCR was used to analyze methylation in the *MdMYB1* promoter, and was performed as described by Telias et al. [11] and Wang et al. [29]. Genomic DNA extracted from peels of ‘Granny Smith’ and ‘Golden Delicious’ was subjected to bisulfite conversion using the EZ DNA Methylation-Gold™ Kit (Zymo Research Corp., Irvine, CA, USA). The treated DNA was used as template, and the *MdMYB1* promoter fragments were amplified using ZymoTaq DNA Polymerase (Zymo Research Corp.). The PCR products were cloned into the PMD18-T vector (TaKaRa) and then sequenced. Twenty independent clones of each fragment were sequenced and analyzed with the online software Kismeth [49]. The *MdMYB1* promoter was amplified by 11 pairs of degenerate primers (Table 2), and the methylation level of each fragment was calculated. Statistical differences were examined by Student’s t-tests at the significance levels of *P* < 0.05 (*) and *P* < 0.01 (**).

**Table 2:** Sequences of forward and reverse primers for amplification of apple genes used in the Bisulfite sequencing-PCR analyses

Primer position	Sense Primer (5′-3′)	Anti-sense Primer (5′-3′)
−2026, − 1870^a^	GAAATYGTTYGAAGGTYTAAGG	ACARCAAACACCCAAAATCC
−1898, −1633	TGATTAAAGGGATTTTGGGTGTTTG	AAATARAACCRRACCTATCTCA
−1667, −1287	GTTTGTAAYAGAYTGAGATAGGT	TCCAAAACCCCARATTCTTAT
−1312, −1035	TATGYATAAGAATYTGGGGTTTTGGA	CTTACTTRRTCACATTATACCATC
−1062, −964	YAYYGATGGTATAATGTGAYYAAGT	ACATTATTTTCATARRTRRTTCARCC
−989, −774^a^	GGYTGAAYYAYYTATGAAAATAATG	ARACRCTACACCTAACACATTRCT
−841, −533	TTGTGAAAGYTTAGTGAGTTGAAG	ATTCTCTCCTTTTTTTCCCTCTTCT
−541,-435	GGAGAGAATYYTAYTYYATAAATTAYAAG	CTTTCRCTRCTTTTTCAARTRTT
−434, −188	TTATGGTGGTYAAAGATGTGTGTTGTAA	CAAACACTRACAARTTTAAAACATTCCAACA
−198, −60	GTYAGTGTTTGYTTYTGTGGATATY	TCCARAAARACACCTARCTACC
−51, 106^a^	AGTGGGTAGYAGGYAAAAGA	TCCACTTTCCCTCTCCATRA

^a^Primers modified from Telias et al. [11]. Y = C or T; and R = A or G

## Additional file

Additional file 1: **Table S1.** *MdMYB1* region promoter (2026 bp) analysis using the PLACE Signal Scan Search database. (DOCX 16.9 kb)

## Abbreviations

5-aza-dC: 5-aza-2′-deoxycytidine
ANS: Anthocyanidin synthase
bHLH: Basic helix-loop-helix
CHI: Calconeisomerase
CHS: Chalcone synthase
DABR: Days after bag removal
DAFB: Days after full bloom
DAFT: Days after exposure of 5-aza-dC-treated fruits to light
DFR: Dihydroflavonol 4-reductase
F3H: Flavonone 3-hydroxylase
UFGT: UDP-glycose: flavonoid 3-O-glycosyltransferas
