# Supplementary material for: The effect of promoter methylation on MdMYB1 expression determines the level of anthocyanin accumulation in skins of two non-red apple cultivars
Source: BMC Plant Biol. 2018 Jun 5;18:108. doi: 10.1186/s12870-018-1320-7 (PMC5989451; doi:10.1186/s12870-018-1320-7)
Supplement: Supplementary file 1 — Table S1. MdMYB1 region promoter (2026 bp) analysis using the PLACE Signal Scan Search database. (DOCX 16.9 kb) [file 12870_2018_1320_MOESM1_ESM.docx]

**Table S1** *MdMYB1* region promoter (2026 bp) analysis using the PLACE Signal Scan Search database

| Motif Name | Location in the Promoter | Sequence | Function |
| --- | --- | --- | --- |
| EECCRCAH1 | -1909, -1517, -1377, -919, -694, -322 | GANTTNC | *MYB*-binding site |
| IBOXCORE | -1078, -446, -8 | GATAA | Conservedsequence upstream of light-regulated genes |
| INRNTPSADB | -1970, -1947, -1833, -1823, -1658, -564 | YTCANTYY | Light-responsive element |
| MYBST1 | -1936, -1185, -180, -229, -129 | GGATA | *MYB*-binding site |
| MYBCORE | -2070, -1873, -1662, -1280, -1243, -857, -222, -219 | CNGTTR | *MYB*-binding site |
| MYBCOREATCYCB1 | -1669, -1597, -1242, -1206 | AACGG | *MYB*-binding site |
| MYBPLANT | -1809, -1274, -789 | MACCWAMC | Plant-*MYB*binding site that regulates phenylpropanoid biosynthesis genes. |
| ABRELATERD1 | -1684, -1490, -1427, -817, -166 | ACGTG | Dehydration-responsive element |
| ACGTATERD1 | -1683, -1489, -1426, -816, -165 | ACGT | Dehydration-responsive element |
| DRE2COREZMRAB17 | -1675 | ACCGAC | Abscisic acid-responsive element |
| LTRECOREATCOR15 | -1674 | CCGAC | Low temperature-responsive element |
| DPBFCOREDCDC3 | -1771, -1620, -471, -272 | ACACNNG | Abscisic acid-responsive element |
| MYB1AT | -1535, -1524, -1179, -1126, -1076, -1014 | WAACCA | *MYB*-binding site |
| CCAATBOX1 | -1371, -1137, -809 | CCAAT | Heat shock-responsive element |
| MYCCONSENSUSAT | -2041, -1370, -1122, -1105, -1010, -924, -470, -222 | CANNTG | *MYC* recognition site |
| GAREAT | -1270, -995 | TAACAAR | GA-responsive element |
| MYB2CONSENSUSAT | -1243, -1207, -857, -222 | YAACKG | *MYB*-binding site |
| CURECORECR | -869, -754 | GTAC | Oxygen-responsive element |
| MYB2AT | -857 | TAACTG | Dehydration-responsive element |
| WBOXATNPR1 | -1790, -1766, -848, -590, -492, -426, -312 | TTGAC | Salicylic acid-responsive element |
| WRKY71OS | -2004, -1789, -1766, -1424, -1046, -1031, -890, -847, -589, -583, -491, -426, -312, -198, -163, -113 | TGAC | Gibberellin-responsive element |
| DPBFCOREDCDC3 | -1771, -1620, -471, -272 | ACACNNG | Abscisic acid-responsive element |
| TATCCAYMOTIFOSRAMY3D | -182, -129 | TATCCAY | Sugar-repression responsive element |
| RYREPEATBNNAPA | -170 | CATGCA | Abscisic acid-responsive element |
| MYCATRD22 | -1010 | CACATG | *MYC*-binding site |
| LTRE1HVBLT49 | -1219 | CCGAAA | Low temperature-responsive element. |
| LTRECOREATCOR15 | -1674 | CCGAC | Core of low temperature-responsive element |

Single underlines, double underlines, and dashed lines indicated the locations of predicted *cis*-acting elements detected within the -541 to -435 bp, -1898 to -1633 bp, and -2026 to -1870 bp regions of the *MdMYB1* promoter, respectively
